# Impairment of Pol β-related DNA base-excision repair leads to ovarian aging in mice

**DOI:** 10.18632/aging.104123

**Published:** 2020-11-20

**Authors:** Ke Hua, Liping Wang, Junhua Sun, Nanhai Zhou, Yilan Zhang, Feng Ji, Li Jing, Yang Yang, Wen Xia, Zhigang Hu, Feiyan Pan, Xi Chen, Bing Yao, Zhigang Guo

**Affiliations:** 1 Jiangsu Key Laboratory for Molecular and Medical Biotechnology, College of Life Sciences, Nanjing Normal University, Nanjing 210023, China; 2 Center of Reproductive Medicine, Jiaxing Maternity and Child Health Care Hospital, College of Medicine, Jiaxing University, Jiaxing 314000, China; 3 Center of Reproductive Medicine, Jinling Hospital, Clinical School of Medical College, Nanjing University, Jiangsu 210002, China; 4 School of Life Sciences, Nanjing University, Nanjing 210093, China

**Keywords:** ovarian aging, BER, Pol β, menopause, oocytes

## Abstract

The mechanism underlying the association between age and depletion of the human ovarian follicle reserves remains uncertain. Many identified that impaired DNA polymerase β (Pol β)-mediated DNA base-excision repair (BER) drives to mouse oocyte aging. With aging, DNA lesions accumulate in primordial follicles. However, the expression of most DNA BER genes, including APE1, OGG1, XRCC1, Ligase I, Ligase α, PCNA and FEN1, remains unchanged during aging in mouse oocytes. Also, the reproductive capacity of Pol β+/- heterozygote mice was impaired, and the primordial follicle counts were lower than that of wild type (wt) mice. The DNA lesions of heterozygous mice increased. Moreover, the Pol β knockdown leads to increased DNA damage in oocytes and decreased survival rate of oocytes. Oocytes over-expressing Pol β showed that the vitality of senescent cells enhances significantly. Furthermore, serum concentrations of anti-Müllerian hormone (AMH) indicated that the ovarian reserves of young mice with Pol β germline mutations were lower than those in wt. These data show that Pol β-related DNA BER efficiency is a major factor governing oocyte aging in mice.

## INTRODUCTION

The decline in reproductive performance with age and decreased conception rates remain challenges in women’s reproductive health. This reduced reproductive performance and oocyte quality closely relates to the decline in ovarian follicle numbers. Furthermore, the nonlinear decline in ovarian reserve indicates that out of the 1 million oocytes at birth, only roughly 500 gets released during reproductive years. This nonlinearity accelerates from age 1-3 and results in almost complete exhaustion by 51-52 years of age on average [1–3]. If the unknown mechanism of attrition acceleration gets identified in later reproductive ages, reproductive outcomes may improve, and the onset of menopause may slow down through targeted intervention. Some research data have indicated that declining ovarian follicle reserve depletion has the potential to prevent menopause from occurring until the age of 71 [1].

In mammalian oocytes, homologous recombination (HR) repair plays a prime role in safeguarding both oocyte quantity and quality [4]. Previous studies on germinal vesicle (GV) oocytes have focused on the role of DNA double-strand break (DSB) repair in ovarian aging [5]. DSBs as the most severe form of DNA lesions can result in carcinogenesis, the apoptotic death of cells, or the onset of cellular senescence [6]. ΓH_2_AX (a marker of DSBs) presents an increasing trend with increasing age [5]. Women bearing mutations in the BRCA genes have been found to exhibit reduced ovarian reserves, with higher rates of DNA damage occurring in the primordial follicle oocytes, however further evidence is still needed [6].

Our previous findings have suggested that a single DNA strand lesion is associated with ovarian aging. GV oocytes remain in the diplotene stage, whereas HR plays a significant role in the transition from the leptotene stage to the pachytene stage [7]. HR-repaired oocytes can be particularly susceptible to genomic damage, with it’s potential to remain dormant in humans for more than 40 years until being stimulated, leading to growth and fertilization [8]. During the period of dormancy, cumulative DNA damage can arise in these oocytes, reflecting the necessity that they can repair DNA to ensure that sufficient oocytes are available for reproduction [8]. When DNA damage in these cells remain unrepaired, mutations can potentially be passed to offsprings, leading to hereditary diseases [8].

Lesions in individual DNA strands can be removed via excision repair processes in which complementary DNA is used to replace individual excised DNA strands. Small mutations arising from errors such as uracil incorporation or oxidative damage and not influencing the overall DNA helical structure can be repaired via the base-excision-repair (BER) process [9].

A landmark genome-wide association study (GWAS) includes an epidemiological analysis of the age of natural menopause involving 70,000 women providing evidence for strong links between DNA BER and the age of natural menopause in the human population [10]. Moreover, the relationship between BER and aging has been an increasing research topic in many tissues and organs, especially female germline, for example, measuring changes in apurinic/apyrimidinic sites (AP sites) levels and kinetics after DNA damage [11]. Senescent human fibroblasts and leukocytes from old donors exhibit more AP sites at baseline than younger cells [12]. H_2_O_2_ or methyl methanesulfonate (MMS) treatment increases the number of AP sites more rapidly in young cells than in old cells, causing a significant decrease in DNA glycosylase activity [13]. Previous studies have assessed the kinetics level of oxidized guanine (8-OHdG) in mouse genomic DNA, and found that aging mice irradiated by γ-irradiation have a higher accumulation of 8-OHdG in their tissues than younger mice [14]. Many reports have observed a reduction in the abundance of DNA polymerase β (Pol β) in mouse and rat’s brain extracts with aging [15].

Although the mechanism of ovarian reserve exhaustion related to the age of natural menopause remains unknown, several pieces of evidence indicate that BER changes (BER activity declines) over the course of aging, with these shifts in functionality likely influencing oxidative DNA damage accumulation and mutation with age. Here, we used wild type (wt) and Pol β heterozygous mice to explore the molecular mechanism of Pol β in ovarian aging at the cellular and animal levels.

Our findings strongly suggested that Pol β plays a central role in natural menopause.

## RESULTS

### Pol β-deficient mouse ovarian function is decreased, and impaired BER leads to ovarian aging

We established Pol β^+/-^ mice as a model for this study (Figure 1A) as Pol β-deficient homozygous mice (Pol β^-/-^) are not viable as previously reported [16]. Tails from Pol β^+/-^ mice had lower Pol β gene expression than tails from wt mice (Figure 1B; P<0.001). Compared to wt mice, Pol β^+/-^ mice showed two lines in PCR nucleic acid gel electrophoresis (Figure 1C). At first, we observed the importance of intact Pol β function in maintaining ovarian reserves in mice by performing a “natural experiment”. Anti-Müllerian hormone (AMH) is a serum biomarker offering the means of estimating primordial follicle reserves and predicting the age at which menopause occurs [17]. Serum AMH concentrations were prospectively compared in wt mice (6-8 weeks), wt mice (8 months) and Pol β^+/-^ mice (6-8 weeks). Mice carrying mutations had markedly reduced levels of AMH in the serum relative to wt mice (6-8 weeks) (Figure 1D; P<0.001).

**Figure 1:**
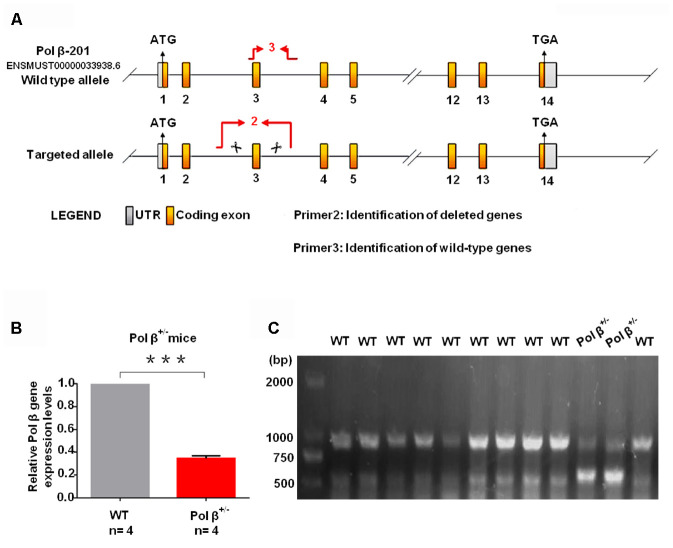
**Pol β-deficient mouse ovarian function and impaired BER lead to ovarian aging.** (**A**) The modeling strategy of pol β^+/-^ mice. (**B**) Relative Pol β gene expression levels in Pol β-deficient mice. We observed significant Pol β deficiency in heterozygous Pol β^+/-^ mice compared with that in wt mice (mice aged 6-8 weeks, n stands for the number of mice, ***P<0.001, Student’s t test). (**C**) To confirm that the Pol β fragment was deleted correctly, total RNA was extracted and Pol β cDNA was amplified via PCR.

**Figure 1:**
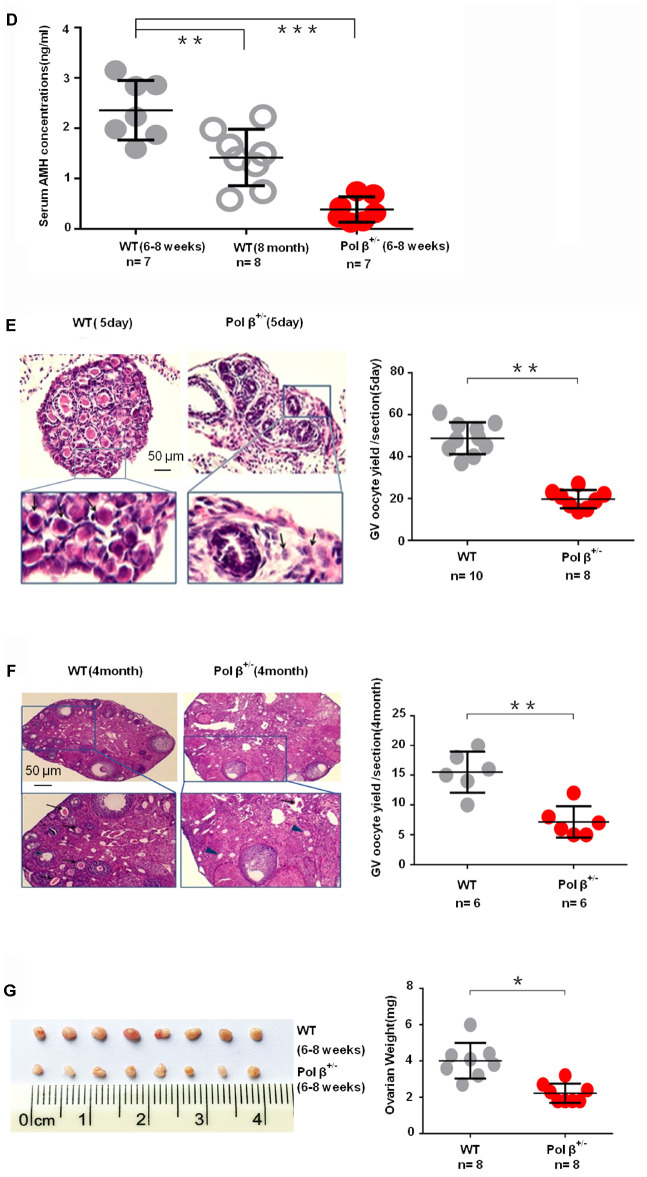
**Pol β-deficient mouse ovarian function and impaired BER lead to ovarian aging.** (**D**) Pol β^+/-^ mice had lower mean serum AMH concentrations than wt mice (aged 6-8 weeks) (n stands for the number of mice, ***P < 0.001, ANOVA). (**E**) Reduced primordial follicles per ovary (5-day ovary; n stands for the number of mice, **P < 0.01; Student’s t test); the black arrow indicates oocytes. (**F**) Reduced primordial follicles per ovary (4-month ovary; n stands for the number of mice, **P < 0.01, Student’s t test); the black arrow indicates oocytes; the blue arrow indicates no oocytes. (**G**) Reduced ovarian weight in Pol β^+/-^ mice (Mice aged 6-8 weeks; n =8 per group; n stands for the number of ovary, *P < 0.05, Student’s t test).

**Figure 1:**
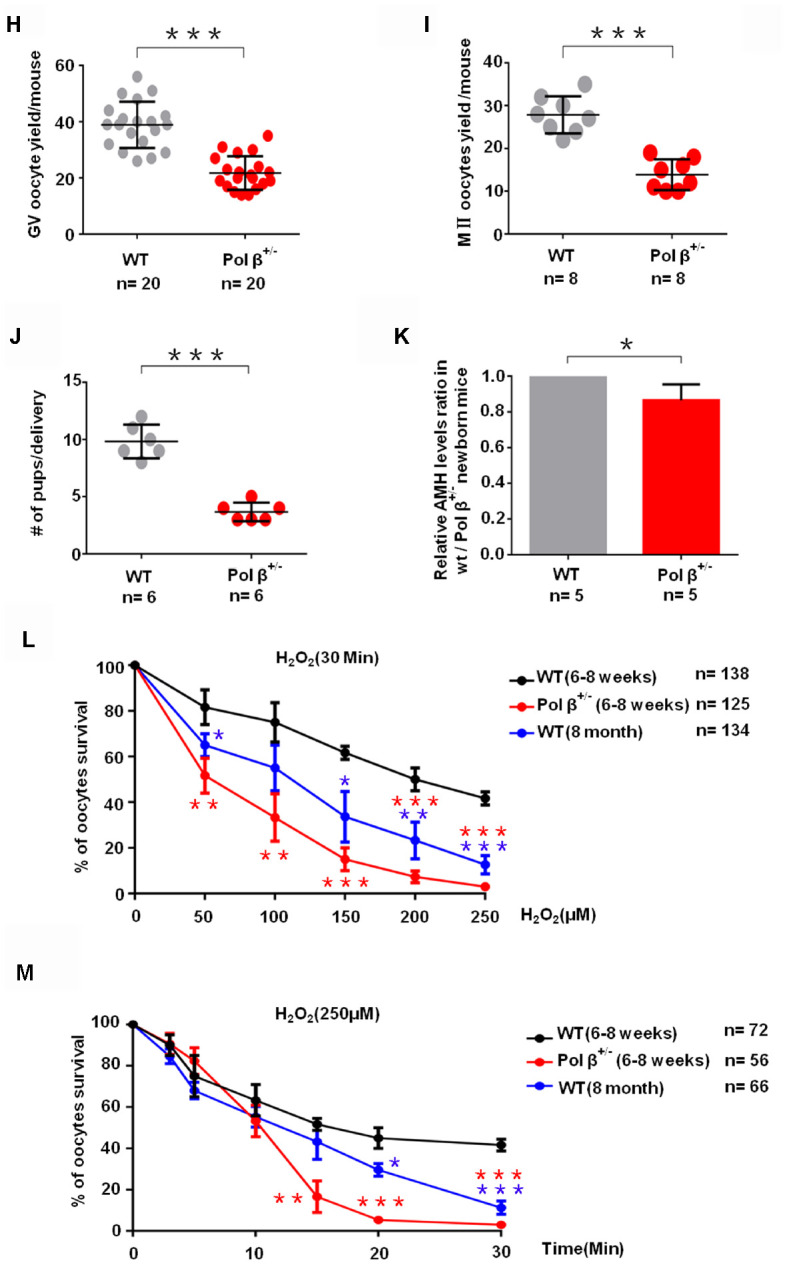
**Pol β-deficient mouse ovarian function and impaired BER lead to ovarian aging.** (**H**) Scatter graphs show a lower number of oocytes yield in Pol β^+/-^ mice compared to wt mice, (Mice aged 6-8 weeks; n =20 per group; n stands for the number of mice, ***P < 0.001, Student’s t test). (**I**) Pol β^+/-^ mice also showed significantly lower meiosis II (MII) oocyte yield per female than wt mice (Mice aged 6-8 weeks; n =8 per group; n stands for the number of mice, ***P<0.001, Student’s t test). (**J**) Reduced litter size in Pol β^+/-^ mice (Mice aged 6-8 weeks, n =6 per group; n stands for the number of mice, ***P < 0.001, Student’s t test). (**K**) In newborn mice, Pol β^+/-^ offspring had lower mean serum AMH concentrations than wt mice (Mice aged 2 weeks, n =5 per group; n stands for the number of mice, *P < 0.05, Student’s t test). (**L**) Compared to wt (6-8 weeks), Pol β^+/-^ (6-8 weeks) and wt (8 months) oocytes after treatment with different H_2_O_2_ concentrations at 30 minutes exhibited different survival (n stands for the number of oocytes, ***P<0.001, **P<0.01, Student’s t test). (**M**) Compared to wt (6-8 weeks), Pol β^+/-^ (6-8 weeks) and wt (8 months) oocytes after treatment with same H_2_O_2_ concentration exhibited different survival (n stands for the number of oocytes, ***P<0.001, ***P<0.001, Student’s t test).

**Figure 1:**
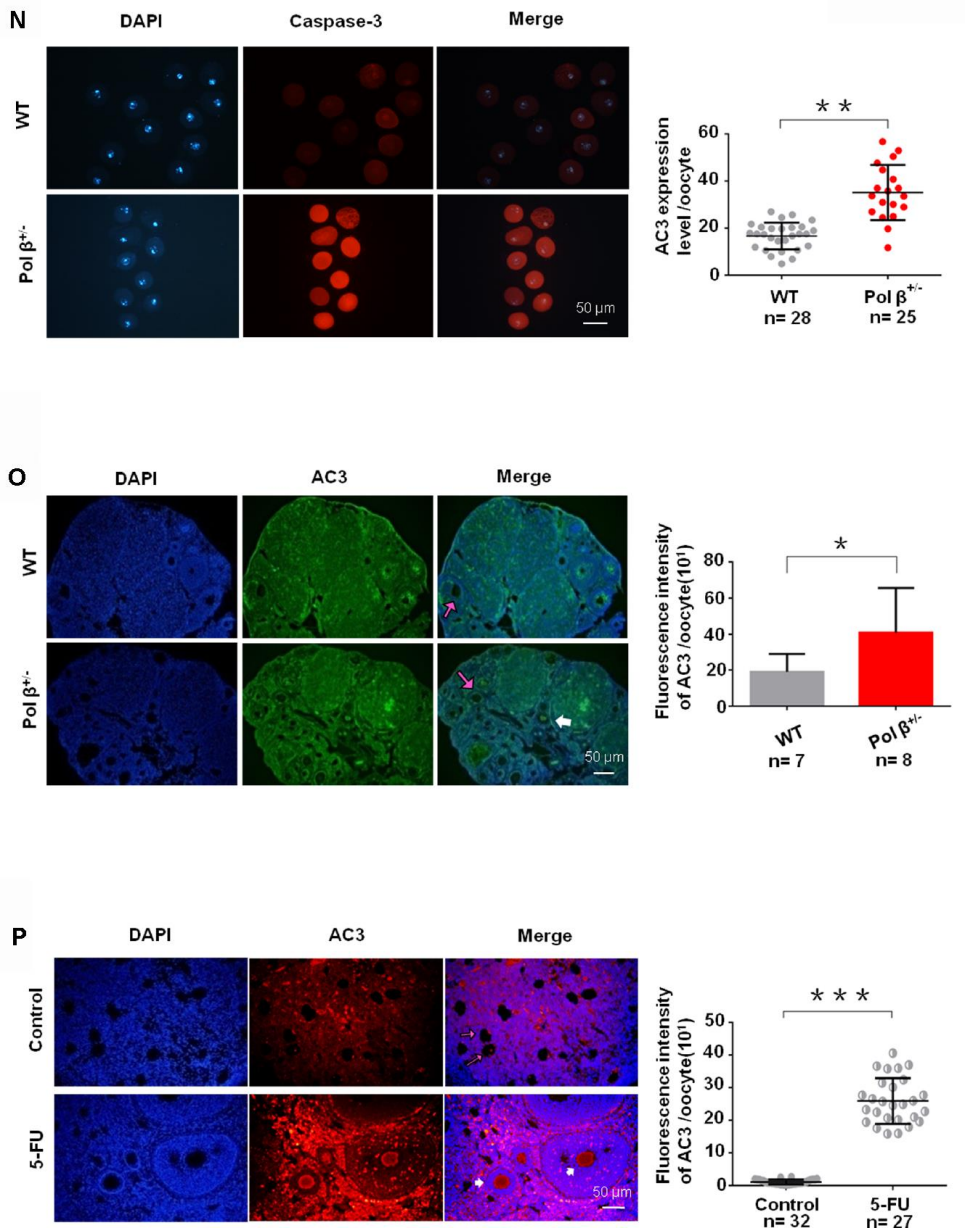
**Pol β-deficient mouse ovarian function and impaired BER lead to ovarian aging.** (**N**) Pol β^+/-^ (6-8 weeks) and wt (6-8 weeks) oocytes were immunofluorescent for AC3. Pol β^+/-^ showed a significant increase in AC3 fluorescence intensity compared to wt (n stands for the number of oocytes, **P<0.01, Student’s t test). (**O**) Pol β^+/-^ (6-8 weeks) ovarian tissue showed a higher AC3 fluorescence intensity (n stands for the number of oocytes, *P < 0.05, Student’s t test). The pink arrow indicates normal oocytes; the white arrow indicates apoptotic oocytes. (**P**) 5-FU treatment results in ovarian tissue with greater AC3 fluorescence intensity (mice aged 6-8 weeks, n stands for the number of oocytes, ***P <0.001, Student’s t test). The pink arrow indicates normal oocytes; the white arrow indicates apoptotic oocytes (mice injected with 5-FU for 2 weeks under the safe dose of 20 mg/kg every day, Student’s t test). All scatter graphs and bar graphs show the means ± SD.

Similar to the AMH result, 5-day-old and 4-month-old Pol β^+/-^ mice exhibited reduced primordial follicle numbers per ovary compared to wt mice of the same age (Figure 1E; P<0.01, 1F; P<0.01, respectively). Pol β^+/-^ mice had lower ovarian weights than wt mice (Figure 1G; P<0.05). Pol β^+/-^ mice released fewer oocytes (GV/ MII) (MII: the metaphase of second meiosis) upon ovarian stimulation than wt mice, with smaller litter sizes after mating (Figure 1H; P<0.001, 1I; P<0.001, 1J; P<0.001, respectively). In newly born mice, Pol β^+/-^ offsprings had lower mean serum AMH concentrations than wt mice (Figure 1K, 2 weeks aged mice, n=5 per group; P<0.05).

Next, we observed that Pol β^+/-^ oocytes showed a significant decline in survival (the difference between the alive and dead oocytes is based on refraction) after treatment with different concentrations of H_2_O_2_ (Figure 1L; P<0.001). In addition, Pol β^+/-^ oocytes exhibited a significant decline in survival after treatment with the same concentration of H_2_O_2_ for different treatment times (Figure 1M; P<0.001). Pol β plays a chief role in BER, which repairs DNA damage and prevents cells from undergoing apoptosis. Furthermore, Pol β^+/-^ oocytes showed significantly increased activated caspase3 (AC3; a marker of apoptosis) than wt oocytes (Figure 1N and 1O; P<0.01, P<0.05, respectively).

Pol β is a prime enzyme for BER, and Pol β- deficiency causes a decline in BER. We administered an intraperitoneal injection of 5-fluorouracil (5-FU; a structural analogue of uracil and thymine specifically targeted by BER) to wt mice to further demonstrate the importance of the balance of DNA damage and BER to sustain ovarian survival. By adding 5-FU to break the balance between DNA lesions and BER, we confirmed that genotoxic stress caused by BER *in vivo* promotes oocyte apoptosis. 5-FU is incorporated into RNA and DNA, resulting in DNA damage. Ovarian tissues treated with 5-FU showed significantly higher fluorescence intensity of AC3 than untreated tissues (Figure 1P; P<0.001), which is consistent with previous studies reporting the role of BER in the cellular/ *in vitro* response to 5-FU [18].

These findings reveal a role for Pol β in the survival of oocytes, suggesting that the BER process is the key to maintain both genome integrity and ovarian reserves.

### Age reduces BER efficiency through low Pol β expression in mouse oocytes

Pol β deficiency leads to oocyte apoptosis, and age leads to a decrease in the ovarian reserve. However, whether Pol β deficiency is related to ovarian aging or not, it is still unknown. C57BL/6J mice, with clearly reduced ovarian reserves over eight months of age (old) compared to 6-8 weeks of age (young), were used to test this hypothesis. Eight months old C57BL/6J mice corresponding to women in the late third decade of reproductive lifespan when both the quantity and quality of oocytes occurred markedly decrease [19].

**Figure 2:**
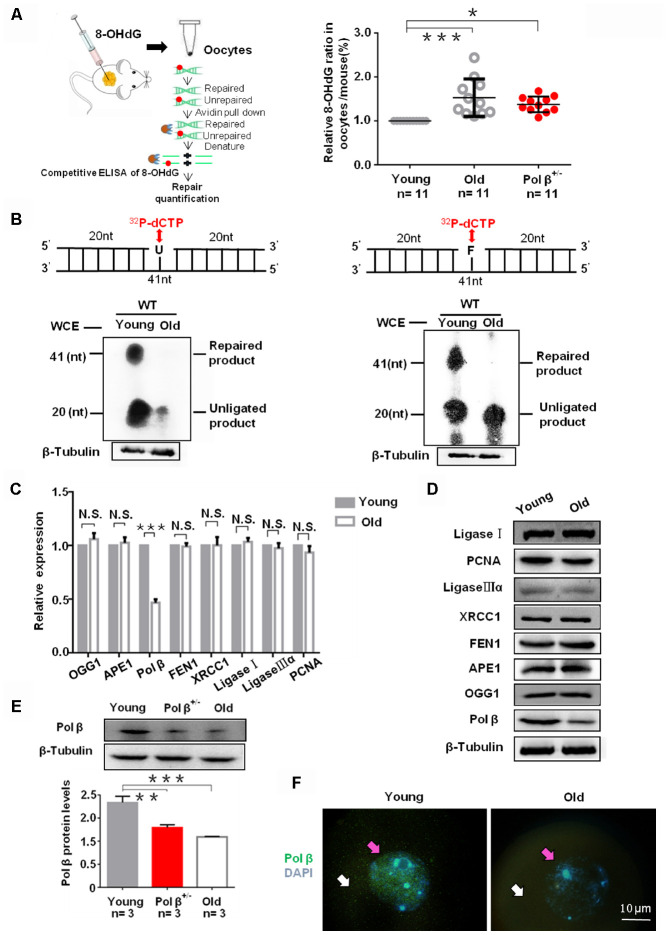
**Aging reduce BER efficiency by low expression of Pol β in mouse oocytes.** (**A**) Three equal dose of DNA oligo containing the damaged DNA lesion 8-OHdG was respectively transfected into young/old Pol β^+/-^ mice ovary; 4h later, oocytes were lysed, and released 8-OHdG was determined by ELISA. With increasing age, reduced 8-OHdG repair was detected, indicating that age inhibits endogenous BER. Scatter graphs represent the content of 8-OHdG in oocytes, with a significant increase in the content of 8-OHdG in old (8 months) / Pol β^+/-^ mice (6-8 weeks) compared to that in young mice (6-8 weeks) (n=11 per group; n stands for the number of mice, ***P<0.001, *P<0.05). (**B**) young/old oocytes, which were respectively divided into two parts for western blotting and BER assays, are defective in BER repair efficiency using whole-cell extract. The number of total oocytes in each line of the gel was detected to be approximately 500 and approximately 700, respectively. SP-BER: reconstitution with young and old oocytes. LP-BER: reconstitution with young and old oocytes. In this assay, whole young and old oocyte extracts were prepared to test SP-BER and LP-BER. Uracil or THF lesions were efficiently repaired by young oocytes but not by old oocytes. (**C**) Significant decrease in the expression of DNA repair genes in old mice (8 months) compared to that in young mice (6 to 8 weeks) shown by qRT-PCR. All results are the mean ± SD (n = 4 per group). Bar graphs represent the gene expression levels. The bar graphs show significantly lower levels of expression for Pol β in old mice than in young mice (***P < 0.001, Student’s t test). (**D**) Significant decrease in the expression of Pol β in old mice (8 months) compared to that in young mice (6 to 8 weeks) shown by western blotting, whereas, no significant difference in other genes. (**E**) Significant decrease in the expression of Pol β in old (8 months) / Pol β^+/-^ mice (6-8 weeks) compared to that in young mice (6 to 8 weeks) shown by western blotting. We also show the quantitation with error bars (n=3 per group; n stands for the number of mice, *** P<0.001, **P<0.01). (**F**) Significant decrease in the expression of Pol β in old mice (8 months) compared to that in young mice (6 to 8 weeks) shown by photomicrographs. Representative photomicrographs show lower amounts of Pol β (green) protein expression in old mice than in young mice. Oocytes were counterstained with DAPI (blue). White arrows point to the cytoplasm and pink arrows to the nucleus. All bargraphs show the means ± SD.

ROS can drive oxidative damage in the DNA, with 8-OHdG as an intermediate product of oxidative damage that is repaired by BER. With increasing age, the detection of reduced 8-OHdG repair, indicated that aging inhibits endogenous BER (Figure 2A; P<0.001) [20]. Besides, Pol β^+/-^ mice (6-8 weeks) also exhibited lower 8-OHdG repair (Figure 2A; P<0.05).

The reduced repair efficiency in old oocytes may link with decreased BER efficiency. We utilized oocyte extracts to assay short-patch BER (SP-BER) and long-patch BER (LP-BER). SP- and LP-BER substrates, utilized uracil-containing (Pol β-U) and tetrahydrofuran-containing (Pol β-F) substrates, respectively. When uracil-DNA glycosylase (UDG) and apurinic/ apyrimidinic endonuclease 1 (APE1) cleaved these substrates, nick forms in the DNA, and ^32^P-dCTP or additional deoxynucleotides, resulting in the formation of short non-ligated intermediate segments. Phosphorimaging can detect these segments. Such intermediates are processed further, yielding a final 41 nucleotide (nt) long repaired product. In the present study, uracil and THF lesions underwent efficient repair in young oocytes but not in old ones. Small ^32^P-dCTP quantities incorporated in DNA substrates and the young oocytes reconstituted further SP- and LP-BER reactions than old oocytes (Figure 2B) [21].

As observed in assays of polymerase activity (Figure 2A and 2B), the reduced BER efficiency in old oocytes was likely due to the low expression of key BER proteins. To assess why DNA damage more readily accumulates in older oocytes, we utilized qRT-PCR to analyze individual oocytes and found significantly reduced Pol β levels in old mice as compared to young mice (Figure 2C; P<0.001). However, we observed no significant difference in the expression patterns of 8-oxoguanine DNA glycosylase (OGG1), APE1, Flap endonuclease 1 (FEN1), X-ray repair cross complementing 1 (XRCC1), Ligase I, Ligase IIIα, and proliferating cell nuclear antigen (PCNA) (Figure 2C; P=0.0785, P=0.351, P=0.5861, P=0.9656, P=0.1128, P=0.3204 and P=0.0705, respectively). Furthermore, western blotting and immunofluorescence showed aging reduced Pol β expression (Figure 2D–2F).

### Aging-related ovarian reserve decreases and single strand breaks (SSBs) from young and old mice

Ovarian sections of young and old mice were immunostained with hematoxylin-eosin to count the number of primordial follicle oocytes.

**Figure 3:**
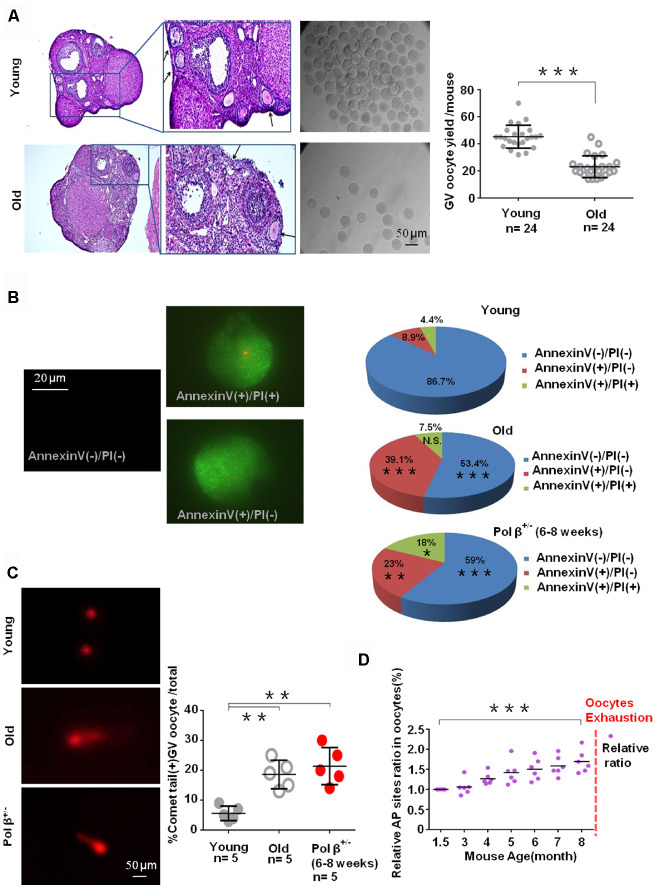
**Aging-related ovarian reserve decline and SSBs from young and old mice.** (**A**) Photomicrographs represent hematoxylin-eosin staining of young (upper) and old (below) C57BL/6J mice ovarian sections. Inset (upper left corner of young mice ovarian tissue) shows a higher number of oocytes yield; scatter graphs show a higher number of oocytes yield in young (6 to 8 weeks) compared to old (8 months) mice, (n =24 per group; n stands for the number of mice, ***P < 0.001, Student’s t test. The experiment was repeated six times). The black arrow indicates oocytes in ovarian follicles. (**B**) Immunofluorescence graphs show a result of Annexin V/PI staining. Statistics of oocytes number per young/ old/ Pol β^+/-^ mouse including Annexin V(-)/PI(-), AnnexinV(+)/PI(+) and Annexin V(+)/PI(-). Adjacent pie charts show old/ Pol β^+/-^ group more Annexin V(+)/PI(-), more Annexin V(+)/PI(+) and less Annexin V(-)/PI(-) compared to young group (6 to 8 weeks), (n=3 per group; ***P<0.001, ** P<0.01, *P<0.05). (**C**) The alkaline comet experiment shows that old/ Pol β^+/-^ oocytes have more percentage of positive comet tail. (n=5 per group; n stands for the number of mice, **P<0.01, **P<0.01, Student’s t test). (**D**) Scatter graphs represent the relative ratio of AP sites in oocytes, with a significant increasing tendency in the content of AP sites in every age group with the increase of age (n=5 per group; ***P<0.001, Student’s t test).

Old mice had significantly fewer oocytes than young mice (Figure 3A; P<0.001). In parallel, we utilized Annexin V-FITC/ propidium iodide (PI) immunofluorescence. A higher percentage of cells underwent early apoptosis in old mice than in young mice (Figure 3B; P<0.001). This study also observed a similar finding in Pol β^+/-^ mice (Figure 3B; P<0.01).

The comet assay, which is a form of electrophoretic analysis of single cells, allows individual analysis of DNA damage within a given cell. In this assay, an alkaline reaction condition (pH>13) identifies genotoxic agents and detects SSBs and alkali-labile sites more efficiently [22]. In the present study, old oocytes and Pol β^+/-^ oocytes had more comet tails than young oocytes (Figure 3C; P<0.01, P<0.01).

Furthermore, the hydroxyl radicals produced as a consequence of the Fenton reaction in response to H_2_O_2_ and superoxide anions can result in several different changes to DNA, with resultant free base release leading to breaks in DNA strands modified by various sugars, as well as AP sites. An AP site is one of the primary forms of damage induced by ROS [11]. In the present study, oocytes had an increasing tendency of AP sites with increasing age (Figure 3D; P<0.001).

### Effects of Pol β knockdown on oocyte apoptosis

**Figure 4:**
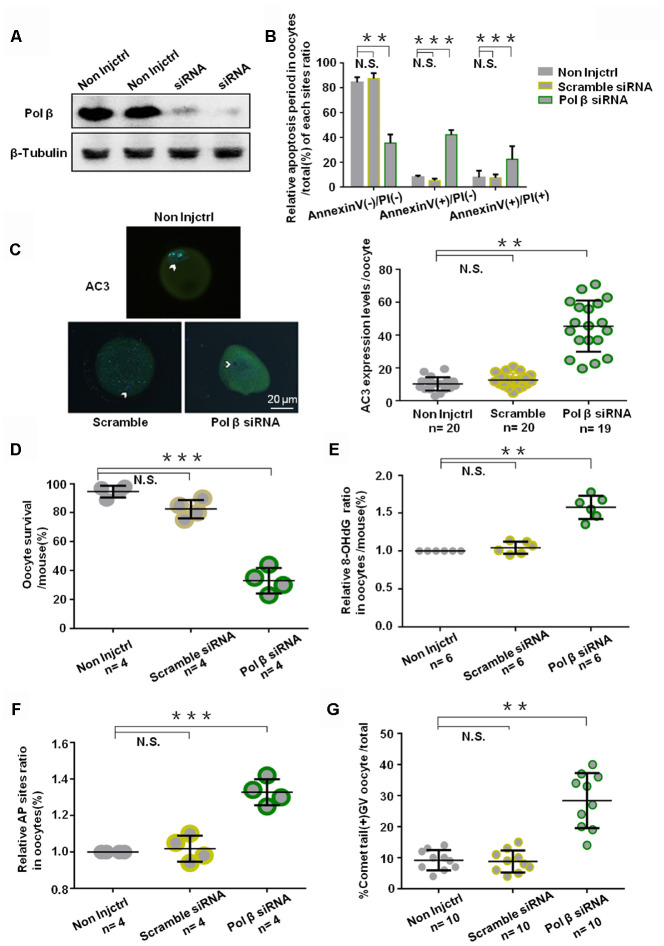
**Effects of Pol β knockdown on oocytes apoptosis.** (**A**) Western blotting analysis indicates that Pol β siRNA group have barely expression. (**B**) Bar graphs show a result with Annexin V/PI staining. Statistics of oocytes number per non-Injctrl/ Scramble siRNA/ Pol β siRNA oocytes including Annexin V(-)/PI(-), AnnexinV(+)/PI(+) and Annexin V(+)/PI(-). Bar graphs show Pol β siRNA group more Annexin V(+)/PI(-), more Annexin V(+)/PI(+), and less Annexin V(-)/PI(-) compared to non Injctrl/ Scramble siRNA group, (n=4 per group; n stands for the number of mice, ***P<0.001; ***P<0.001; **P<0.01, respectively). (**C**) Impact of siRNA silencing of Pol β on genomic integrity and survival of mouse oocytes in response to genotoxic stress. In response to H_2_O_2_ treatment (250 μM), AC3 levels were higher (n stands for the number of oocytes, **P < 0.01, Student’s t test). Photomicrographs are representative of the AC3 levels in the siRNA-silenced oocytes: scramble, Pol β (green). Arrowheads show the nuclear region. Oocytes are counterstained with DAPI (blue). (**D**) Survival was lower in the oocytes in which the expression of Pol β had been silenced compared to those in controls (scramble siRNA) (n stands for the number of mice, ***P < 0.001). (**E**) Scatter graphs represent the 8-OHdG in oocytes, with a significant increase in Pol β siRNA oocytes compared to that in non-Injctrl/ scramble siRNA oocytes (n=6 per group; n stands for the number of mice, **P<0.01). (**F**) Scatter graphs represent the relative ratio of AP sites in oocytes, with a significant increase in Pol β siRNA oocytes compared to that in non-Injctrl/ scramble siRNA oocytes (n stands for the number of mice, ***P<0.001, Student’s t test). (**G**) Scatter graphs represent the percentage of positive comet tail in oocytes, with a significant increase percentage of positive comet tail in Pol β siRNA oocytes compared to that in non-Injctrl/ scramble siRNA oocytes (n stands for the number of mice, **P<0.01, Student’s t test). Each experiment was repeated three times. All graphs show the means ±SD.

To assess the specific role of Pol β in oocytes and to analyze the importance of BER in protecting oocyte genomic integrity and survival, we injected the young murine oocytes with small interfering RNA (siRNA) targeting Pol β and the “All Stars Negative Control siRNA” as a negative control. Western blotting (WB) confirmed that oocytes with Pol β siRNA showed downregulated Pol β expression (Figure 4A). Annexin V-FITC/propidium iodide (PI) immunofluorescence identified oocytes undergoing Pol β siRNA-induced apoptosis. Immunofluorescence analysis showed a marked decrease in normal oocytes and a significant increase in apoptotic oocytes (Figure 4B; P<0.01, P<0.001 and P<0.001, respectively). Genotoxic stress then induced in these siRNA-treated oocytes via H_2_O_2_ treatment. All oocytes with siRNA-downregulated gene expression exhibited a significantly increased fluorescence intensity of AC3 after H_2_O_2_ treatment than scramble siRNA-treated oocytes, indicating that interference with Pol β results in an increased apoptotic signaling activity in oocytes (Figure 4C; P<0.01). After 24 hours, oocytes treated with Pol β siRNA exhibited a lower survival rate than the non-injection control (non-injctrl) or scramble siRNA treated oocytes (Figure 4D; P<0.001). Figure 4E (Figure 4E; P<0.01), shows a significant increase in 8-OHdG observed in Pol β siRNA-treated oocytes compared to non-injctrl or scramble siRNA-treated oocytes, causing DNA lesion accumulation at the intracellular level. Figures 4F and 4G show significant increases in the AP sites and percentages of comet tails in Pol β siRNA-treated oocytes compared to non-injctrl or scramble siRNA-treated oocytes, suggesting that interference with Pol β results in increased DNA damage at the intracellular level (Figure 4F and 4G; P<0.001, P<0.01, respectively). These results revealed that the Pol β-related DNA BER pathway is critical for oocyte survival. Almost all non-injctrl oocytes survived under the identical conditions.

### Pol β overexpression ameliorates maternal age-associated ovarian reserve exhaustion.

**Figure 5:**
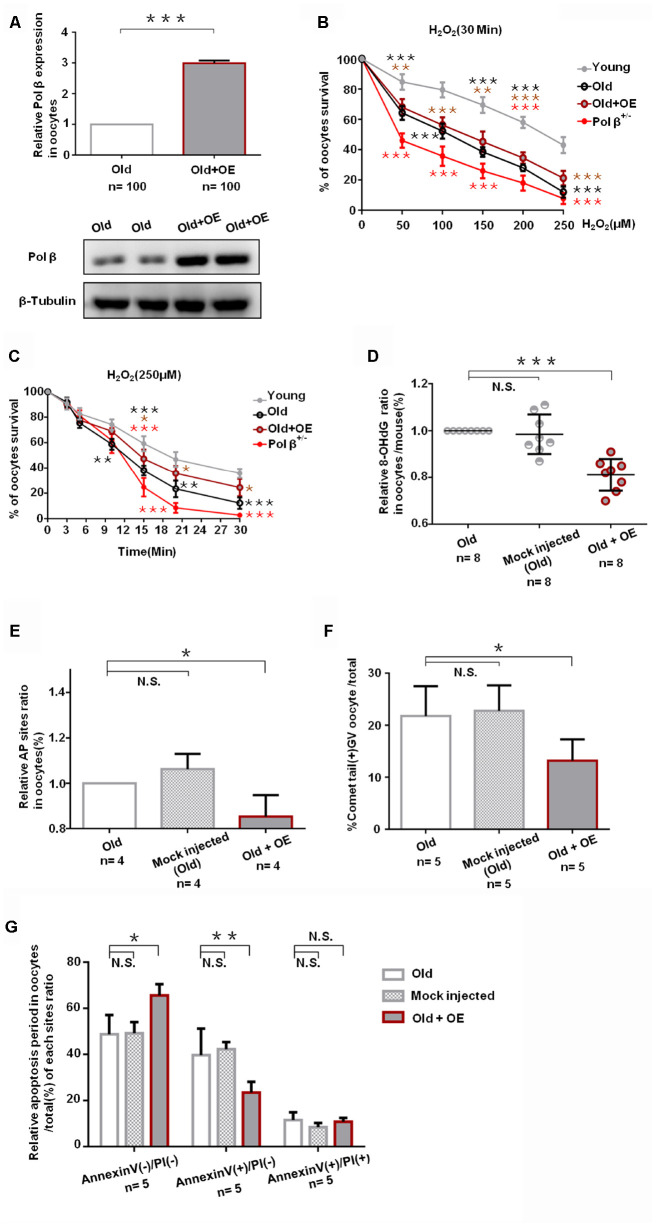
**Pol β overexpression ameliorates maternal age-associated ovarian reserve exhaustion.** (**A**) Relative Pol β gene expression levels in Pol β OE. We observed significant Pol β expression increase compared to old mice by WB and QPCR (mice aged 8 months, n stands for the number of oocytes, ***P<0.001, Student’s t test). (**B**) Microinjection of the Pol β plasmid in response to genotoxic stress. Compared to wt (6-8 weeks), wt (8 months) / Pol β OE (8 months) / Pol β^+/-^ (6-8 weeks) oocyte after treatment with different H_2_O_2_ concentrations at 30 minutes exhibited different survival. We observed a significant increase in survival in overexpressed oocytes compared to old mice (***P<0.001, Student’s t test). (**C**) Microinjection of the Pol β plasmid in response to genotoxic stress. Compared to wt (6-8 weeks), wt (8 months) / Pol β OE (8 months) / Pol β^+/-^ (6-8 weeks) oocyte after treatment with the same H_2_O_2_ concentration exhibited different survival. We observed a significant increase in survival in overexpressed oocytes compared to old mice (*P<0.05, Student’s t test). (**D**) Scatter graphs represent the 8-OHdG in oocytes, with a significant decrease in Pol β OE oocytes compared to that in old/ mock injected oocytes (n=8 per group; n stands for the number of mice, ***P<0.001). (**E**) Bar charts represent the relative ratio of AP sites in oocytes, with a significant decrease in Pol β OE oocytes compared to that in old/ mock injected oocytes (n stands for the number of mice, *P<0.05, Student’s t test). (**F**) Bar charts represent the percentage of positive comet tail in oocytes, with a significant decrease percentage of positive comet tail in Pol β OE oocytes compared to that in old/ mock injected oocytes (n stands for the number of mice, *P<0.05, Student’s t test). (**G**) Bar charts show an result with Annexin V/PI staining. Statistics of oocytes number per old/ mock injected/ Pol β OE oocytes including Annexin V(-)/PI(-), AnnexinV(+)/PI(+) and Annexin V(+)/PI(-). Bar charts show Pol β OE oocytes more Annexin V(-)/PI(-), and less Annexin V(+)/PI(-) compared to that in old/ mock injected oocytes except Annexin V(+)/PI(+) (n=5 per group; n stands for the number of mice, *P<0.05; **P<0.01, respectively).

We exposed mock-injected (injected with empty plasmid) and Pol β complementary DNA (cDNA) -injected oocytes from aged murine to H_2_O_2_ in order to test whether Pol β overexpression (OE) increases genotoxic stress resistance or not. Oocytes from Pol β-OE mice showed more Pol β gene expression than wt mice by QPCR and WB (Figure 5A; P<0.001), and Pol β-OE oocytes showed a significant increase in oocyte survival after treatment with different concentrations of H_2_O_2_ (Figure 5B; P<0.001). Also, Pol β-OE oocytes exhibited a significant increase in oocyte survival after treatment with the same concentration of H_2_O_2_ for different lengths of time (Figure 5C; P<0.05). A decrease in 8-OHdG was observed in Pol β-OE oocytes compared to old or mock-injected oocytes, suggesting that Pol β-OE enhances BER to resist DNA oxidative damage (Figure 5D; P<0.001). Figures 5E and 5F show significant decrease in AP sites and percentages of positive comet oocytes in Pol β-OE oocytes compared to old or mock-injected oocytes (Figure 5E; P<0.05, 5F; P<0.05, respectively). These results showed a significant increase in normal oocytes and a significant decrease in apoptotic oocytes, suggesting that restoration of the Pol β function may prolong the life time of aging oocytes (Figure 5G; P<0.05, P<0.01, respectively).

### Diminished ovarian reserve in Pol β -deficient mice

**Figure 6:**
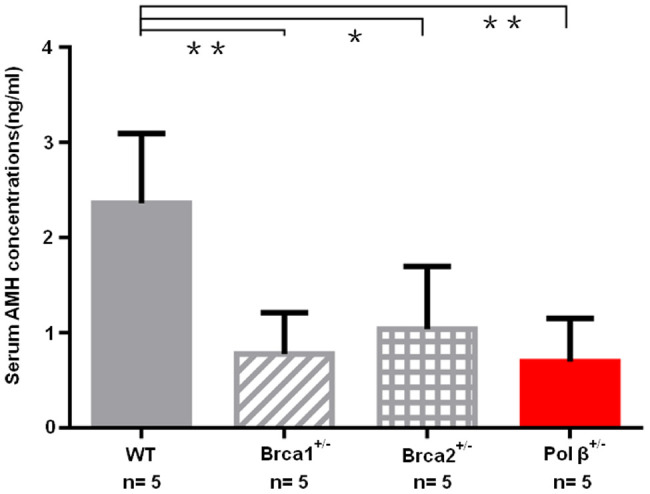
**Diminished ovarian reserve in Pol β-deficient mice.** Mice with BRCA1 (**P <0.01) or BRCA2 (*P <0.05) mutations had significantly lower mean serum AMH concentrations; Pol β^+/-^ mice had significantly lower mean serum AMH concentrations compared to wt mice (**P < 0.01, Student’s t test), similarly to mice with BRCA1 or BRCA2 mutations. All bar graphs show the mean ± SD. (N stands for the number of mice).

Because breast cancer 1 (Brca1) and breast cancer 2 (Brca2) associates with ovarian aging [5, 23], we prospectively compared serum AMH concentrations in wt mice, Brca1^+/-^ mice, Brca2^+/-^ mice and Pol β^+/-^ mice at the same age. Pol β^+/-^ mice displayed significantly lower serum concentrations of AMH than wt mice, but similar serum concentrations of AMH with Brca1^+/-^ mice and Brca2^+/-^ mice (Figure 6; P<0.01, P<0.01 and P<0.05, respectively).

## DISCUSSION

The free radical theory of aging asserts that oxidative damage will gradually accumulate over time, leading to the deterioration of cell function and inducing an aging phenotype. BER is a key DNA repair pathway responsible for repairing damaged DNA caused by oxidative stress, and is often thought to prevent cellular senescence. Therefore, it may be involved in the aging process. Here, we identified a new link between the key BER enzyme Pol β and oocyte senescence *in vivo* and *in vitro*.

In meiosis prophase I, oocytes experience the leptotene, zygotene, pachytene, diplotene and diakinesis stages. GV oocytes remain in the diplotene stage, whereas HR plays a chief role in the transition from the leptotene stage to the pachytene stage [4], where a time difference exists. HR exclusively repairs DNA double-strand breaks [7]. GV oocytes (HR-repaired oocytes) can be particularly susceptible to genomic damage, given their potential to remain dormant in humans for more than 40 years until being stimulated, leading to growth and potential fertilization [8]. During the period of dormancy, cumulative DNA damage can arise in these oocytes, making it capable of repairing DNA to ensure that sufficient oocytes are available for reproduction. But if the DNA damage does not get repaired in time, the mutations that occur will spread to the next generation and lead to their genetic diseases [5, 8]. Our findings suggest that a single DNA strand lesion is associated with ovarian aging.

BER begins with a particular DNA glycosylase, which excises the improper base from the DNA, and later APE1 cuts into the backbone of the DNA molecules to yield an intermediate nicked abasic structure that can then undergo immediate SP-BER or LP-BER [24–26]. In SP-BER, Pol β adds a single base to the 3’-end of this nicked site, after Pol β mediates the 5’-sugar phosphate residue, followed by β-elimination via its deoxyribose phosphate (dRP) lyase functionality. This event results in the development of a nick which can subsequently undergo repair by XRCC1/Ligase III α [27, 28]. In LP-BER, Pol β or Polδ mediate strand displacement synthesis, wherein a 2-10 nucleotide DNA flap gets produced and subsequently excised through the activity of FEN1 [29–33]. DNA ligase seals Nick I [26]. When any of these prime genes remain knocked down, inevitably leading to reduced BER functionality and increased DNA damage accumulation, thereby leading to the death of cells, thus explaining the observed decrease in oocyte survival.

The Pol β-deficient mouse model showed more rapid ovarian follicular reserve depletion as well as SSB accumulation in mouse oocytes. Single-cell qRT-PCR uncovered Pol β as a vital enzyme for DNA BER in ovarian aging. RNAi studies revealed that Pol β related DNA BER pathways were essential for oocyte survival and maintaining DNA integrity. Mouse data identified Pol β deficiency as a critical means of mediating oocyte aging in mammals.

Pol β heterozygous knock out (k/o) mice have lower Pol β expression in somatic tissues such as the brain, lungs and other organs. Although our research focused on ovarian reserve, we reviewed some reports which demonstrated a reduction in the abundance of Pol β in mouse and rat brain extracts with aging [34, 35]. Oocytes and sperm are terminally differentiated cells that no longer divide and require DNA repair to resist DNA damage caused by endogenous and exogenous factors to avoid death. Pol β is a key DNA repair protein. So it appears to be more important to oocytes than in divisible cell such as hepatocytes. As well, in other species such as zebrafish and chicken Pol β also play an important role on sustaining cell survival [36, 37].

Other findings associated with senescence diseases of BER support the present study and demonstrate a relationship between BER and aging. Proteins that bind to telomeres can interact with a variety of DNA repair enzymes, highlighting the key role of DNA repair in telomeres, which are highly vulnerable to oxidative damage, with BER being the primary means of repairing such damage [38]. A recent meta-analysis of GWASs of single-nucleotide polymorphisms identified numerous potential DNA repair genes associated with menopause [39]. Pol β deficiency is known to disrupt the efficiency of BER, leading cells to be more sensitive to agents causing alkylating and oxidative stress [40]. When Pol β gets knocked out in mice, BER remains non-functional, leading to hypersensitivity of mutant cells to agents that can cause DNA damage including methyl methanesulfonate (MMS) and N-methyl-N-nitrosourea (MNU), that drive rapid embryonic lethality [16]. Moreover, any mutations that interfere with the ability of Pol β to mediate its dRP lyase, or polymerase activities, or to interact with specific proteins, can impair *in vitro* BER activity [41]. Pol β mutation carriers may also be born with lower ovarian reserves, as observed in the newborn Pol β heterozygous mice in this report (Figure 1B). Pol β is essential to the DNA oxidative damage response, and prolonged oxidative damage is known to drive ovarian aging [42]. Therefore, impaired Pol β activity can lead to more rapid oogonia and oocyte attrition.

As the ovarian reserve and fertility decline with age, higher rates of pregnancy failure and more frequent occurrences of errors in meiosis that lead to abnormal chromosomal separation and subsequent conception abnormalities are evident with increasing age [43]. The specific driver of decreased oocyte quality with age remains unclear, although Pol β is essential for mouse meiotic synapsis [44]. During prophase I, Pol β is localized in the synaptonemal complex (SC), and during zygonema and pachynema, it localizes to synapsed axes, and then interacts with the ends of bivalents in late pachynema and diplonema [44]. Pol β expression links to an age-related decrease in male germ cells in the same species [45]. Additionally, BER, which is associated with oxidative DNA damage caused by aging, might also play a role in anti-ovarian aging [46], and the frequency of SSBs increases with age [47].

Thus, we were able to incorporate our results with those from previous studies to propose a unified model (Supplementary Figure 1) of how aging drives oocyte both quality and quantity decrease. Principally, we recommend a model in which the efficiency of DNA repair decreases with age, with increasing accumulation of SSBs and higher numbers of oocytes eliminated to prevent severe mutations from passing to offsprings. As BER genes get expressed at lower levels and this expression rapidly drops during the latter portion of the third decade of life, this event leads to accelerating drops in the quantity and quality of oocytes. BER is consistent with previous models of how the shortening of telomeres influences oocyte aging in a process linked to the impaired ability of DNA BER mechanisms to maintain telomere integrity [48]. Furthermore, epigenetic phenomena declining in DNA BER with age will likely bring fascinating insights into ovarian aging, which, in turn, may facilitate development of future strategies for bolstering the efficiency of DNA repair, thereby slowing of ovarian aging, and the delaying of menopause.

## MATERIALS AND METHODS

### Materials

The chemicals and media used were obtained from Sigma-Aldrich; Merck KGaA (Darmstadt, Germany) unless stated otherwise. C57BL/6J mice were used in this study. The Ethics Committee of College of life science laboratory animal center, Nanjing normal university approved all animal studies.

### Mouse oocyte preparation

GV oocytes from young (6 - 8 weeks), old (8 months) and C57BL/6J mice (6 - 8 weeks) (corresponding to women aged 51 whose ovarian reserve exhausted) were collected and liberated from surrounding cells by brief treatment with hyaluronidase (200 IU/ml) in M199 medium at room temperature. Regarding oocyte RNA isolation, cells were first separated and suspended in PBS, whereas cells used for immunofluorescence underwent 4% paraformaldehyde (PFA) fixation. Further, ovaries from old and young animals were isolated, fixed, paraffin embedded, and cut into 8 μm thick sections for immunohistochemistry (IHC).

### IHC and immunofluorescence

Regarding Pol β and AC3 staining, oocytes were fixed for 30 minutes in 4% paraformaldehyde (PFA) and then treated for 20 minutes with 0.5% Triton X-100 to permeabilize cells at room temperature. Samples were then blocked for 1h using PBS containing 1% BSA and then probed with the following antibodies: Anti- Pol β and anti- AC3 at 4° C overnight. Samples were then washed three times using PBS, stained for 1h with AF488 goat-anti rabbit IgG, and then stained using DAPI (blue) to detect nuclei at room temperature. The anti-fade medium (Vectashield, CA, USA) was used to mount samples, which were then assessed under a laser scanning confocal microscope (LSM710, Zeiss, Germany) at 40x. The software and version are NIS-Elements Viewer (v4.2.0).

### Fluorescence quantification

Z stacks of 8 μm thick sections of each sample were collected via microscopy, and the resultant combined image with maximal signal intensity was imported into ImageJ (NIH, Bethesda, MD, USA) and used for quantitative analyses. When measuring AC3 staining intensity, an entire oocyte was selected, and then ImageJ determined the mean AC3 intensity within each oocyte.

### Single oocyte qRT-PCR

Oocytes that had been frozen using PBS (Solarbio) were thawed and lysed in an appropriate lysis buffer (9803S; Cell Signaling), and then two RNA amplification cycles were conducted using the Sensation RNA Amplification kit (SNSAT12; Genisphere). An RNeasy Mini kit (74104; Qiagen) was used to isolate RNA, and a NanoDrop 1000 (Thermo Scientific) was used to gauge RNA quality and quantity, whereas Experion capillary electrophoresis (Bio-Rad) was used to gauge RNA integrity. A total of 1 mg of amplified RNA from individual samples was reverse transcribed using appropriate N9 primers (Vazyme), dTVN (Vazyme), and Superscript III reverse transcriptase (Invitrogen). SYBR Green (Vazyme) was used on an Applied Biosystems 7300 Real-Time PCR device for qRT-PCR, with the ΔΔCt and ΔCt methods used to compare old and young oocyte gene expression, with β-Actin (Sangon) used for normalization. The sequence of the primers used for the qRT-PCR were listed in Table 1.

**Table 1:** REAGENT or RESOURCE used in the study.

**REAGENT or RESOURCE**	**SOURCE**	**IDENTIFIER**
Antibodies		
rabbit anti-Pol β	Abclonal	Cat#A1681
rabbit anti-FEN1	Abclonal	Cat#A0129
mouse anti-APE1	Abcam	Cat#13B8E5C2
rabbit anti-Active Caspase-3	R&D Systems	Cat#AF835
Cy3 Goat Anti-Mouse IgG	Abclonal	Cat#AS008
Cy3 Goat Anti-Rabbit IgG	Abclonal	Cat#AS007

### Transgenic mouse analyses

Pol β^+/-^ transgenic animals were selected based on their genotoxic stress sensitivity [16] and obtained from the Model Animal Research Centre of Nanjing University, China. Animals were housed in a standard climate-controlled facility under a 12-hour light/dark cycle. These mice carry a 365 bp deletion in exon 3 (aaaagcggcatctgtgatagccaagtacccacacaaaatcaagagcggagcggaagctaagaaactg) of Pol β (Pol β^+/-^) induced via CRISPR/Cas9 editing. Homozygous Pol β^-/-^ mice are not viable, dying at embryonic day 10.5, whereas heterozygous Pol β^+/-^ embryos displayed both chromosomal abnormalities and slower post-implantation growth [16].

### Genotyping

We conducted genotyping using DNA obtained from ear/tail biopsies based on standard protocols. Briefly, samples were lysed in a buffer containing proteinase K (Invitrogen) at 55° C overnight. Then, an equal quantity of isopropanol was used for DNA precipitation. The pellet was washed using 70% ethanol, air dried, resuspended in nuclease-free water, and then used for PCR together with appropriate primers (Forward:CGTGCTGGAAAGGCAAATCT/ Reverse:CCTTCAGAAAGACTGCCAGC) for genotyping.

### Mating

Housing females were mated with heterozygous males at a 2:1 ratio. Upon reaching 3 weeks of age, pups were weaned. wt and Pol β^+/-^ mouse mating rates were determined based on detection of vaginal plugs, which revealed comparable fecundity rates for both groups of animals.

### AMH ELISA

A standard curve was prepared for each plate, with a high performance elisa (HPE) buffer. For sample analysis, each sample was diluted with HPE buffer (1:41 ratio). 50 μl of this diluted sample was then added to each of the two wells in a plate that had been pre-coated with the F2B12/H antibody. Plates were incubated for 2 h, and then washed in PBST, and 50 μl of biotinylated monoclonal F2B7/A per well was added (1:3000 in 1% casein) for 1h. The samples were then washed 5 times and incubated for 30 minutes with an HRP conjugate (1:20000 in 1% casein). The plates were then washed once each in PBST and dH2O (distilled H2O), and then tetramethylbenzidine (TMB) substrate was added for 10 minutes while the plates were protected from light. Next, 100 μl per well of 6% phosphoric acid was added to terminate the reaction, and absorbance at 450nm and 655nm was measured using a microplate reader (i-control^TM^1.11, TECAN). The experiment was used by commercial kit (MEIMIAN, Cat. No.MM-44204M2).

### Histomorphometric analyses

After ovarian sections (8 μm) were prepared as described above, they were mounted onto Super Frost Plus glass slides followed by hematoxylin and eosin staining. Next, primordial follicles (with a single layer of flattened granulosa cells) were counted, allowing calculation of total follicle numbers based on a previous report [49].

### RNA interference

Fully grown GV oocytes were microinjected with between 5-10 pl Pol β siRNA (50 μM, GenePharma) or Pol β cDNA(1 mg/ml, GenePharma) via Eppendorf FemtoJet (Eppendorf AG) while being observed under a Leica inverted microscope (M50, Kramer Scientific) along with an Eppendorf TransferMan NK2 micromanipulator. Injected oocytes were grown using HTF media containing 0.4% bovine serum albumin (BSA) for 8 h, followed by treatment with 250 μM H_2_O_2_ for 5 min at 4° C, and then oocytes were washed three times for 2 min each using fresh human tubal fluid (HTF). Oocytes were transferred into fresh HTF and grown under paraffin oil at 37° C in a 5% CO_2_ incubator. As controls, oocytes were instead microinjected with 5 to 10 pl of “All Stars Negative siRNA” from GenePharma (50 μM). Oocyte survival was assessed based on established morphological criteria [50]. The oocytes that survived were stained for AC3 and assessed via microscopy.

### Alkaline comet assay

Oocytes from young (6 - 8 weeks) and old mice (8 months) were treated with 250 μM H_2_O_2_ for 1 h and then washed in PBS and grown using fresh media for 24h. Comet assays were conducted as in previous studies [51]. Oocytes were embedded in 20 μl of low-melting-point agarose(0.5% in dH_2_O at 37° C) on dry slides coated using (1.5% gelatin in PBS) followed by submersion in chilled lysis buffer (1% Triton, 2.5M NaCl, 100 mM EDTA, 10 mM Tris-Hcl, and 1% Na-laurylsarcosine, pH=10) for 1 h. Denaturation and equilibration of the slides were performed for 20minutes using chilled running buffer (300 mM NaOH and 1 mM EDTA, pH>13), and then the samples underwent electrophoresis at 4° C at 0.8 V/cm (300 mM for the alkaline version) for 22 minutes. For alkaline versions of this assay, water, was used to rinse slides that were then fixed using 100% ethanol, before drying and propidium iodide staining (50 μg/ml). Fluorescent microscopy was used to image the slides. Samples were in triplicate using the above procedure.

### Base excision repair assay

The experiment method referred to previous reports [52]. Pol β SP-BER activity and LP-BER activity were assessed with a U and F-containing synthetic DNA duplex (41 nucleotides long). BER reactions were conducted using 20 μl of reaction buffer (40 mM HEPES-KOH (pH 7.8), 70 mM KCl, 7 mM MgCl_2_, 1 mM dithiothreitol, 0.5 mM EDTA, 2 mM ATP, 50 μM each of dATP, dTTP and dGTP, and 8 μM ^32^P-dCTP) volume. To assess SP-BER and LP-BER, sample lysates were combined with appropriate substrates (Pol β-U and Pol β-F) respectively for 30 minutes at 37° C. An equal volume of gel-loading buffer was then added to terminate reactions prior to autoradiography mediated visualization. Note that in SP-BER\ LP-BER substrate, radio-labeled dCTP can only be incorporated in the second position next to the damaged base. The oligonucleotide substrate details were provided in Table 2.

**Table 2:** Oligonucleotides used in the study.

**Oligonucleotides**		
Mouse Endogenous Reference Genes Primers	This publication	B661302
APE1 Genes Primers	This publication	N/A
Forward: GTGCCTCCAAGAGACCAAGTG		
Reverse: TCTTCCTCGCCAATGCCATAAG
Pol β Genes Primers	This publication	N/A
Forward: CGTGCTGGAAAGGCAAATCT		
Reverse: CCTTCAGAAAGACTGCCAGC		
FEN1 Genes Primers	This publication	N/A
Forward: GGCATGTTCTACCGTACCATCC
Reverse: TGAACTTCTCCACCTCCTCCTC

SP-BER substrate Pol β –U (5′-CTTACACGTTGACTACCTTTUTTTGAGGAAAGAGTGGATGG-3′/3′-GAATGTGCAACTGATGGAAAGAAACTCCTTTCTCACCTACC-5′) and the LP-BER substrate Pol β-F (5′-CTTACACGTTGACTACCTTTFTTTGAGGAAAGA GTGGATGG-3′/3′-GAATGTGCAACTGATGGAAAGAAACTCCTTTCTCACCTACC-5′).

### *In vivo* 8-OHdG BER assay

Biotin-conjugated oligonucleotides containing an 8-OHdG lesion capable of undergoing LP-BER repair were used for this assay [53]. This substrate was a biotin-tagged double-stranded oligonucleotide conjugated to an unlabeled strand with one 8-OHdG lesion. Three equal doses of DNA oligo containing the damaged DNA 8-OHdG were respectively transfected into young (6 - 8 weeks) /old (8 months) /Pol β^+/-^ (6 - 8 weeks) mouse ovaries, oocytes were transfected with this DNA and incubated for 4 h, and then the cells were lysed, and biotinylated substrate molecules were isolated with magnetic streptavidin beads. Denaturation was then used to release collected biotinylated DNA, which was then quantified and utilized in a competitive 8-OHdG ELISA using a commercial kit (Trevigen, Cat. No.4380-192-K).

### Western blotting

Laemmli sample buffer supplemented with protease inhibitors was used to lyse approximately 500 oocytes. Samples were then heated to boiling for 5 minutes and separated via 10% SDS-PAGE before transfer to PVDF membranes that were subsequently blocked with 5% non-fat milk in TBST for 1 h. Blots were then probed overnight with the following antibodies at 4° C: Rabbit anti-Rabbit (1:1000) and anti-tubulin (1:1000). Blots were then washed three times with TBST, before probing with secondary HRP-conjugated antibodies for 1 h, and then an ECL Plus Western Blotting Detection System was used for protein band visualization. Band density was quantified using ImageJ. Antibody details were provided in Table 3.

**Table 3:** Oligonucleotide substrate used in the study.

**NAME**	**Oligonucleotide substrate**	**Application**
Pol β-U:		SP-BER with cell lysates
	5′-GCAGGACGAGGGTATCCUACAAAGTCCAGCGTACCATA-3′	
	3′-CGTCCTGCTCCCATAGGATGTTTCAGGTCGCAAGGTAT-5′	
Pol β-F:		LP-BER with cell lysates
	5′-GCAGGACGAGGGTATCCFACAAAGTCCAGCGTACCATA-3′	
	3′-CGTCCTGCTCCCATAGGATGTTTCAGGTCGCAAGGTAT-5′	
SiPol β-mouse- 882:		In cell knockdown assay
	sense:5′- GGAGUGACAUCUUUAAUAATT -3′	
	Anti-sense: 5′- UUAUUAAAGAUGUCACUCCTT -3′	
SiPol β-mouse- 188:		In cell knockdown assay
	sense:5′- GCAUCUGUGAUAGCCAAGUTT-3′	
	Anti-sense: 5′- ACUUGGCUAUCACAGAUGCTT -3′	
8OHdG		In vivo 8OHdG BER assay

### Statistical analyses

Data are the means ± SD. Student’s t-tests and one-way ANOVAs were used to compare data as appropriate, with Prism 6 (GraphPad, CA, USA) used for all comparisons. P<0.05 was the significance threshold.

## Supplementary Materials

Supplementary Figure 1
