# Supplementary material for: Impairment of Pol β-related DNA base-excision repair leads to ovarian aging in mice
Source: Aging (Albany NY). 2020 Nov 20;12(24):25207–28. doi: 10.18632/aging.104123 (PMC7803579; doi:10.18632/aging.104123)
Supplement: Supplementary Figure 1 [file aging-12-104123-s001.pdf]

SUPPLEMENTARY FIGURE

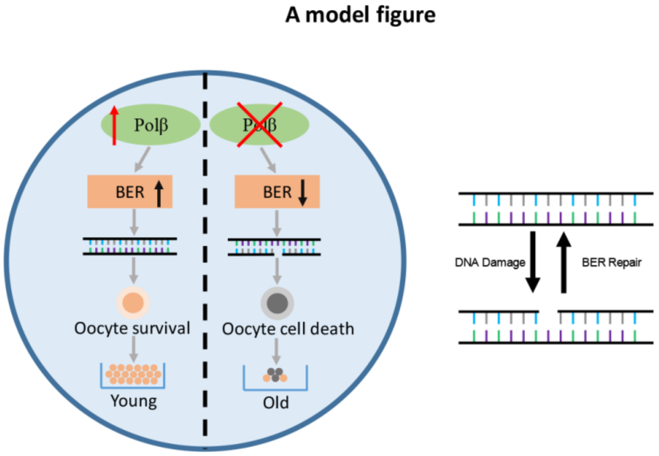

Supplementary Figure 1. Graphical summary of the model for impairment of Pol β- related DNA base-excision repair leads to ovarian aging in mice by this study.
